# An Inverted-Sandwich Diuranium μ-η^5^:η^5^-Cyclo-P_5_ Complex Supported by U-P_5_ δ-Bonding**

**DOI:** 10.1002/anie.201501728

**Published:** 2015-04-27

**Authors:** Benedict M Gardner, Floriana Tuna, Eric J L McInnes, Jonathan McMaster, William Lewis, Alexander J Blake, Stephen T Liddle

**Affiliations:** School of Chemistry, University of NottinghamUniversity Park, Nottingham, NG7 2RD (UK); School of Chemistry and Photon Science Institute, University of ManchesterOxford Road, Manchester, M13 9PL (UK)

**Keywords:** cyclo-P_5_, density functional theory, phosphorus, uranium, *δ* bonding

## Abstract

Reaction of [U(Tren^TIPS^)] [**1**, Tren^TIPS^=N(CH_2_CH_2_NSi*i*Pr_3_)_3_] with 0.25 equivalents of P_4_ reproducibly affords the unprecedented actinide inverted sandwich cyclo-P_5_ complex [{U(Tren^TIPS^)}_2_(μ-η^5^:η^5^-cyclo-P_5_)] (**2**). All prior examples of cyclo-P_5_ are stabilized by d-block metals, so **2** shows that cyclo-P_5_ does not require d-block ions to be prepared. Although cyclo-P_5_ is isolobal to cyclopentadienyl, which usually bonds to metals via σ- and π-interactions with minimal δ-bonding, theoretical calculations suggest the principal bonding in the U(P_5_)U unit is polarized δ-bonding. Surprisingly, the characterization data are overall consistent with charge transfer from uranium to the cyclo-P_5_ unit to give a cyclo-P_5_ charge state that approximates to a dianionic formulation. This is ascribed to the larger size and superior acceptor character of cyclo-P_5_ compared to cyclopentadienyl, the strongly reducing nature of uranium(III), and the availability of uranium δ-symmetry 5f orbitals.

The cyclopentadienyl anion is a ubiquitous ligand in organometallic chemistry,[1] and therefore there is great interest in studying isolobal analogues in terms of overcoming the challenges of preparing them and understanding how they bind to metal ions. One such congener is the cyclo-P_5_ anion, which is of special interest due to the diagonal relationship between carbon and phosphorus.[2] To date, cyclo-P_5_ is found as a formal monoanion in d-block complexes where electron-rich metals promote the formation and stabilization of the P_5_ ring by a number of binding modes to the π-system and phosporus lone pairs, including terminal-η^5^,[3] bridging μ-η^5^-σ^*n*^ or μ-η^5^:η^5^-σ^*n*^,[4] spectacular fullerene-type topologies,[5] or μ-η^5^:η^5^,[6] though the latter mode is less common. Where the f-block is concerned, there are no examples of actinide cyclo-P_5_ derivatives,[7] and only two examples incorporating lanthanide ions are known,[8] which notably both contain the d-block fragments used to construct and introduce the cyclo*-*P_5_ unit. There is, therefore, a question mark over whether the synthesis and stabilization of cyclo-P_5_ by electron-rich d-block metals is a mandatory requirement. Furthermore, given the absence of any actinide cyclo-P_5_ complexes, it is not clear how such a fragment would bind to uranium because although many inverted sandwich arene-C_*n*_ (*n*=4, 6–8) complexes are now known,[9] cyclopentadienyl is conspicuous by its absence. Cyclopentadienyl tends to bind to d-block metals via σ- and π-interactions, and δ-bonding tends to be minimal due to poor spatial overlap from the small size of the cyclopentadienyl ring,[10] but the different frontier orbital energies and larger size and superior acceptor character of cyclo-P_5_, compared to cyclopentadienyl, make predictions impractical. In a wider context, the activation of P_4_, a potential source of cyclo-P_5_, by f-block complexes is incredibly rare,[11] despite widespread interest in the activation of this highly strained pnictide, and cyclo-P_5_ stands out as the missing member of the P_4_ and P_6–8_ family assembled by f-block-promoted catenations to date.

As part of our studies into uranium–pnictide chemistry,[12] we extended our examination of the reactivity of P_4_ with uranium to [U(Tren^TIPS^)] (**1**, Scheme 1; Tren^TIPS^=N(CH_2_CH_2_NSi*i*Pr_3_)_3_). Herein, we report that this gives the actinide cyclo-P_5_ complex [{U(Tren^TIPS^)}_2_(μ-η^5^:η^5^-cyclo-P_5_)] (**2**).

**Scheme 1:**
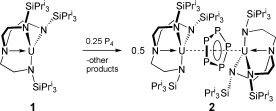
Synthesis of 2 from 1.

Treatment of **1** with a quarter molar equivalent of P_4_ (U:P=1:1) in THF results in the dark blue–green suspension turning brown. After work-up, recrystallization of the sticky brown solid from toluene afforded brown crystals of **2** in 25 % yield (based on phosphorus).[13] Although this yield is low, it is reproducible, and most likely represents the lower degree of orbital control over the phosphorus catenation than compared to the d-block. Indeed, given the prior paucity of actinide cyclo-P_5_ complexes it is remarkable that **2** can be isolated at all. The ^1^H NMR spectrum of **2** spans the range −33 to +9 ppm and the Evans method magnetic moment at 298 K is 4.09 μ_B_; both of these observations are consistent with the presence of two uranium(IV) ions in **2**. Complex **2** is silent its ^31^P NMR spectrum, most likely because of the direct contact between the paramagnetic uranium ions and the cyclo-P_5_ unit. The IR spectrum of **2** contains no absorptions in the P=H stretch region, which is consistent with the absence of a protonated form of the cyclo-P_5_ unit, and the empirical formulation is supported by CHN analyses.

The solid-state structure of **2** was determined by X-ray crystallography and is illustrated in Figure 1.[13] The salient feature of **2** is the presence of a cyclo-P_5_ unit sandwiched between two [U(Tren^TIPS^)] fragments in a μ-η^5^:η^5^ coordination mode. A crystallographic twofold rotation axis runs through one phosphorus center and the mid-way point of a P=P bond on the cyclo-P_5_ ring in the asymmetric unit, and as a consequence the cyclo-P_5_ unit is disordered over two positions. The P=P bonds required restraints during refinement because of the disorder, so no meaningful discussion of the P=P metrical data can be made, but it is clear that the cyclo*-*P_5_ ring is planar in **2**. The U=P bond lengths span the range 3.250(6)–3.335(6) Å, which are longer than the sum of the single-bond covalent radii of U and P (2.81 Å),[14] perhaps reflecting the steric demands of Tren^TIPS^ and the η^5^-bound nature of the cyclo-P_5_ unit. As a strict requirement of residing over the crystallographic twofold rotation axis, the two uranium–Tren fragments are identical, and notably the U=N bond distances are consistent with Tren-ligated uranium(IV) centers,[15] being too short for U^III^=N or averaged U^III/IV^=N distances.[16] An a priori description of **2** would be a mixed valence diuranium(III/IV) with a cyclo-P_5_ monoanion; the crystallographic analysis, however, is inconsistent with this. To investigate this unexpected aspect further we probed complex **2** by spectroscopic and magnetic methods.

**Figure 1:**
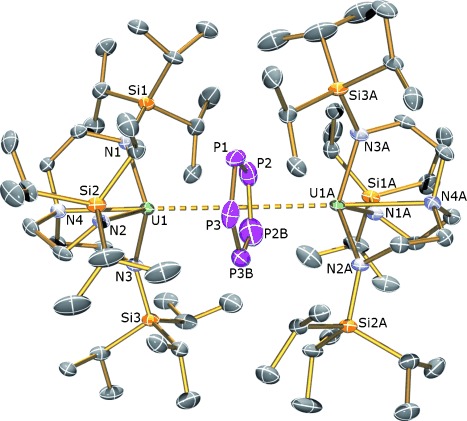
Molecular structure of [{U(Tren^TIPS^)}_2_(μ-η^5^:η^5^-cyclo-P_5_)] (2).[20] Ellipsoids are set at 50 % probability; hydrogen atoms and disorder components are omitted for clarity. Selected bond lengths [Å]: U1–P1 3.335(6), U1–P2 3.243(6), U1–P2B 3.303(7), U1–P3 3.265(7), P3B 3.255(6), U1–N1 2.254(7), U1–N2 2.255(7), U1–N3 2.273(7), U1–N4 2.701(8), P1–P2 1.996(6), P1–P3 2.006(6), P2–P2B 2.002(6), P3–P3B 2.008(8), P2B–P3B 2.018(6).

The UV/Vis/NIR spectrum of **2**[13] exhibits weak (*ε*= <80 L mol^−1^ cm^−1^) absorptions in the range 5000–15 000 cm^−1^ that are characteristic of intraconfigurational, Laporte-forbidden f–f transitions in the ^3^H_4_ manifold of uranium(IV).[17] A charge transfer band tails in from the UV region to about 15 000 cm^−1^, but is of sufficiently low absorbance in the region where any 5f^3^→5f^2^6d^1^ transitions for uranium(III) would occur for them to be visible;[17c,^18^] however, no such absorbances are apparent, and although this does not conclusively rule out **2** containing localized uranium(III) centers it is consistent with a diuranum(IV) formulation. This would, however, invoke a formal dianion formulation for the cyclo-P_5_ unit rather than the more likely monoanionic formulation.

To probe the formal oxidation states of the uranium centers in **2**, we conducted variable-temperature and variable-field SQUID magnetometry measurements on a powdered sample (Figure 2).[13] The effective magnetic moment of **2** is 3.92 μ_B_ at 300 K, which compares well to the Evans method magnetic moment value of 4.09 μ_B_. This corresponds to an effective magnetic moment of 2.77 μ_B_ per uranium ion, which is well within the range for uranium(IV).[19] The magnetic moment decreases fairly monotonously with decreasing temperature, with the decrease being more pronounced below 75 K, reaching 0.99 μ_B_ (0.70 μ_B_ per uranium ion) at 1.8 K. At the same time, *χ* *T*(*T*) decreases from 1.92 cm^3^ K mol^−1^ at 300 K to 0.12 cm^3^ K mol^−1^ at 1.8 K (0.06 cm^3^ K mol^−1^ per uranium ion),[13] supporting the diuranium(IV) formulation of **2**. In agreement with this, there is no hysteresis loop at 1.8 K, and the magnetization shows a steady increase with increasing field reaching 0.48 μ_B_ at 7 Tesla applied field (0.24 μ_B_ per uranium ion). Taken together, these data are consistent with a diuranium(IV) formulation for **2**. In the absence of an analogue of **2** containing diamagnetic metal ions it is currently not possible to determine the cyclo-P_5_ unit contribution, if any, to the magnetic susceptibility of **2**, though we note that the low-temperature magnetic moment per uranium ion is ca 0.2 μ_B_ higher than might be expected,[19] which may reflect a contribution to the magnetic moment from the cyclo-P_5_ ring. Cyclic voltammetry experiments were precluded by the incompatibility of **2** with polar solvents.

**Figure 2:**
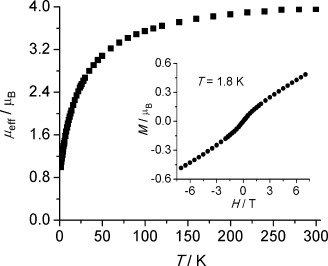
Temperature dependence of the effective magnetic moment of 2 recorded at 0.1 T applied field. Inset: Plot of *M*(H) at 1.8 K.

To further probe the nature of **2**, we conducted a single-point energy calculation on the geometry optimized structure of **2**.[13] The calculated bond lengths and angles are within 0.05 Å and 2° of the experimentally determined structure and so we conclude that the calculated structure represents a qualitative model of the electronic structure of **2**. Interestingly, in the calculation the cyclo-P_5_-ring is slightly puckered,[9] which may be attributed to the gas-phase nature of the calculation compared to the experimental structure of **2** that is subject to crystal packing forces. The calculated MDC_q_ uranium charges are 3.04 and 3.07, which is towards the high end for Tren–uranium(IV) complexes.[12,15] The cyclo-P_5_ unit carries a total charge of −2.68, distributed evenly amongst the five P-centers; this suggests substantial charge transfer from the uranium centers, which is also consistent with the calculated MDC_*m*_ uranium spin densities of −1.93 and −1.98. The latter values are consistent with 5f^2^ uranium(IV) centers, though we note that uranium(IV)–Tren complexes tend to exhibit calculated MDC_*m*_ values of about 2.3 that are consistent with donation of electron density from the ligands to uranium, whereas for **2** uranium is a net exporter of electron density to the cyclo-P_5_ unit. Importantly, we note that the calculated excess spin density on the cyclo-P_5_ unit is −1.69, which suggests significant radical character. The calculated U=P and P=P Mayer bond orders for **2** span the range 0.32–0.60 and 0.68–0.78, respectively, which suggests polarized covalent interactions. For comparison, the U=N_amide_ and U=N_amine_ bond orders average 0.90 and 0.25, respectively. The HOMO, HOMO−1, and HOMO−2 α-spin Kohn Sham frontier orbitals of **2** are each singly occupied and of essentially pure 5f character. HOMO−3 and HOMO−4 (Figure 3) are each singly occupied in the α-spin manifold and represent the principal interactions of the U(P_5_)U unit. These two orbitals result from donation from uranium 5f-orbitals of δ-symmetry into the δ-symmetry e_2_ cyclo-P_5_ frontier molecular orbitals. The HOMO−3 and HOMO−4 are dominated by uranium 5f and phosphorus 3p contributions, being composed of 36/48 and 35/42 % 3p/5f character (that is, ca. 50:50 3p/5f character in each), respectively, with the remainder of each molecular orbital being accounted for by small nitrogen contributions derived from the Tren^TIPS^ ligands. Interestingly, complex **2** appears silent in its powder X-band EPR spectrum at 300, 30, and 5 K, which may reflect the 3p–5f mixing giving rise to efficient relaxation mechanisms. The natures of the five α-spin frontier orbitals of **2** are consistent with the overall occupation of four 5f and one cyclo-P_5_ e_2_ combination. Thus, the calculations are in overall agreement with the combined characterization data and together suggest that the most appropriate description of **2** is that two uranium(III) ions have each singly reduced the cyclo-P_5_ unit to give two uranium(IV) centers with a cyclo-P_5_ charge state that is approaching a dianion, rather than monoanion formulation. The two δ* combinations for the U(P_5_)U unit in **2** are represented by LUMOs +9 and +10, which lie about 1.8 eV higher than HOMO−3 and HOMO−4. This corresponds to a δ–δ* gap of about 14 500 cm^−1^ (690 nm), and an absorption at ca. 690 nm (ε≈85 L mol^−1^ cm^−1^) is observed in the experimental UV/Vis/NIR spectrum of **2**.[13]

**Figure 3:**
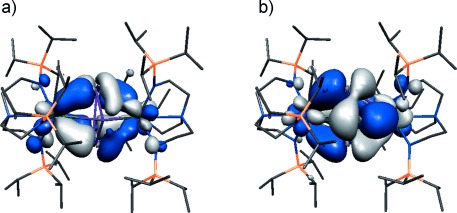
α-spin Kohn Sham orbitals which represent the principal components of the δ-bonding in the U(P_5_)U unit of 2 at the 0.03 e Å^3^ isosurface level. Hydrogen atoms are omitted for clarity. a) HOMO−3 (472a, −4.070 eV); b) HOMO−4 (471a, −4.098 eV).

To conclude, we have reported the synthesis and characterization of the first structurally authenticated actinide cyclo-P_5_ complex. The preparation of this d-block-metal-free complex demonstrates that the cyclo-P_5_ unit can be constructed without d-block ions. However, there is clearly less control over its construction, which results in low yields of **2**. That **2** can be isolated at all is significant given the prior paucity of f-block cyclo-P_5_ complexes, but importantly its isolation permits an analysis of how cyclo-P_5_ can bind to actinide elements. Cyclopentadienyl usually bonds to metals via σ- and π-interactions with minimal δ-bonding, and although cyclo-P_5_ is isolobal to cyclopentadienyl, theoretical calculations suggest the principal bonding in the U(P_5_)U unit is two polarized δ-bonds. This can be attributed to the larger size and superior acceptor character of cyclo-P_5_ compared to cyclopentadienyl and the availability of uranium δ-symmetry 5f-orbitals. Surprisingly, the combined characterization data are consistent with charge transfer from the uranium ions to the cyclo-P_5_ unit such that the charge state of the latter approximates to a dianionic formulation. However, with the presence of δ-bonding and 3p–5f orbital mixing the five frontier α-spin electrons are delocalized across the U(P_5_)U unit. Therefore, given this electronic structure the assignment of oxidation states and spins to individual centers is not clear-cut and we use this electronic structure as a framework in which to rationalize the bonding in **2**, as is the case in inverted sandwich diuranium C_6_-arene complexes.[9] This unexpected outcome, can be attributed to the strongly reducing nature of uranium(III) coupled to the excellent acceptor properties of cyclo-P_5_. Lastly, complex **2** also represents an isolobal analogue of a diuranium inverted sandwich cyclopentadienyl complex, which remains conspicuous by its absence in the burgeoning inverted sandwich arene-C_*n*_ (*n*=4, 6–8) family.[9]

Dedicated to Professor Manfred Scheer on the occasion of his 60th birthday
