# Supplementary material for: An Inverted-Sandwich Diuranium μ-η5:η5-Cyclo-P5 Complex Supported by U-P5 δ-Bonding
Source: Angew Chem Int Ed Engl. 2015 Apr 27;54(24):7068–72. doi: 10.1002/anie.201501728 (PMC4517156; doi:10.1002/anie.201501728)
Supplement: Supplementary file 1 [file anie0054-7068-sd1.pdf]

Angewandte Chemie
Eine Zeitschrift der Gesellschaft Deutscher Chemiker

Supporting Information

German Edition: DOI:

An Inverted-Sandwich Diuranium μ-η^5:η^5-Cyclo-P_5 Complex Supported by U-P_5 δ-Bonding**

*Benedict M. Gardner, Floriana Tuna, Eric J. L. McInnes, Jonathan McMaster, William Lewis, Alexander J. Blake, and Stephen T. Liddle**

anie_201501728_sm_miscellaneous_information.pdf

Experimental

General

All manipulations were carried out using Schlenk techniques, or an MBraun UniLab glovebox, under an atmosphere of dry nitrogen. Solvents were dried by passage through activated alumina towers and degassed before use or were distilled from calcium hydride. All solvents were stored over potassium mirrors except ethers which were stored over activated 4 Å sieves. Deuterated solvent was distilled from potassium, degassed by three freeze-pump-thaw cycles and stored under nitrogen. [U(Tren^{TIPS})] [**1** = Tren^{TIPS} = {N(CH$_2$CH$_2$NSiPr$^i_3$)$_3$}] was prepared as described previously.[1] ^{1}H and ^{29}Si NMR spectra were recorded on a Bruker 400 spectrometer operating at 400.2 and 79.5 MHz respectively; chemical shifts are quoted in ppm and are relative to TMS (^{1}H, ^{29}Si). FTIR spectra were recorded on a Bruker Tensor 27 spectrometer. UV/Vis/NIR spectra were recorded on a Perkin Elmer Lambda 750 spectrometer. Data were collected in 1mm path length cuvettes loaded in an MBraun UniLab glovebox and were run versus the appropriate reference solvent. Static variable-temperature magnetic moment data were recorded in an applied dc field of 0.1 T on a Quantum Design MPMS XL7 superconducting quantum interference device (SQUID) magnetometer using doubly recrystallised powdered samples. Samples were carefully checked for purity and data reproducibility between several independently prepared batches for each compound examined. Care was taken to ensure complete thermalisation of the sample before each data point was measured and samples were immobilised in an eicosane matrix to prevent sample reorientation during measurements. Diamagnetic corrections were applied for **2** using tabulated Pascal constants and measurements were corrected for the effect of the blank sample holders (flame sealed Wilmad NMR tube and straw) and eicosane matrix. X-band EPR data were acquired on a Bruker EMX spectrometer. CHN microanalyses were carried out by Tong Liu at the University of Nottingham. CCDC deposition number for **2** is 1049738.

Preparation of [{U(Tren^{TIPS})}$_2$(μ-η^5:η^5-P_5)] (2)

THF (20 ml) was added to a cold (−78 °C) mixture of [U(Tren^{TIPS})] (**1**) (0.51 g, 0.6 mmol) and P_4 (19 mg, 0.15 mmol). The dark blue-green suspension was allowed to warm to ambient temperature whilst stirring and the dark mixture produced was stirred at room temperature for a further 16 hours. The dark brown solution was reduced to dryness *in vacuo* to afford a sticky dark brown solid, which was extracted into toluene (10 ml), filtered and reduced in volume to *ca*. 2 ml. Storage at 5 °C for 72 hours yielded brown crystals of **2**, which were isolated by filtration, washed with hexanes (3 × 1 ml) and dried *in vacuo* for 30 minutes. Yield: 60 mg (25% based on P). ^{1}H NMR (C_6D_6, 298 K): −32.32 (s, 8H, CH_2), −22.21 (s, 8H, CH_2), −12.68 (s, 8H, CH_2), 5.21 (s, 54H, Me), 6.04 (s, 18H, CH), 8.86 (s, 54H, Me) ppm. ^{29}Si{^{1}H} NMR (C_6D_6, 298 K): −96.87 ppm. ^{31}P signal not observed. μ_{eff} (Evans method, C_6D_6, 298 K): 4.09 μ_B. FTIR (Nujol): 1631.54 (m), 1598.74 (m), 1556.95 (m), 930.86 (s), 670.39 (s), 576.97 (m), 558.70 (m), 541.05 (w), 472.13 (w), 416.37 (w) cm^{-1}. Anal. calc'd for $C_{66}H_{150}N_8P_5Si_6U_2$: C 42.74%; H 8.15%; N 6.04%. Found: C 42.52%; H 8.29%; N 5.77%.

UV/Vis/NIR Spectra

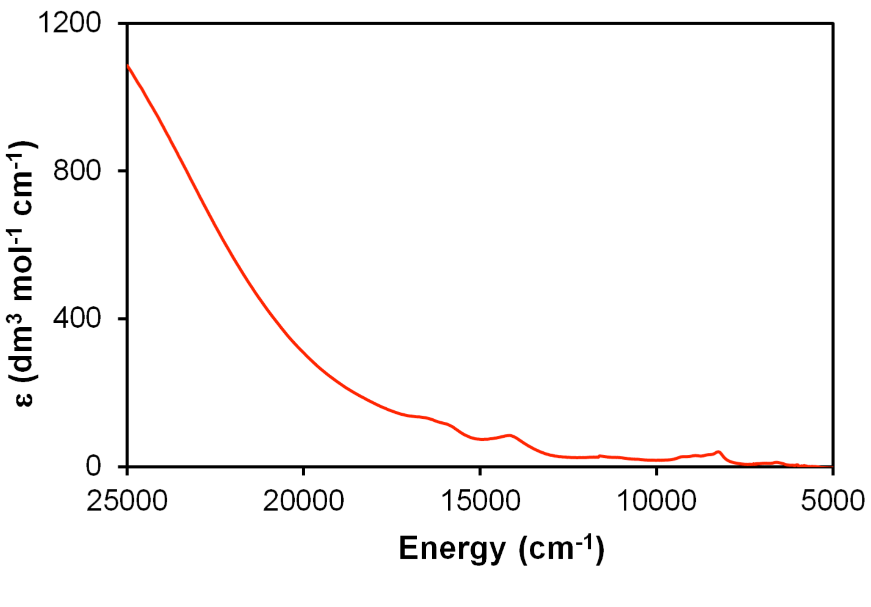

Figure S1. UV/Vis/NIR spectrum of **2** as a 20 mM solution in toluene.

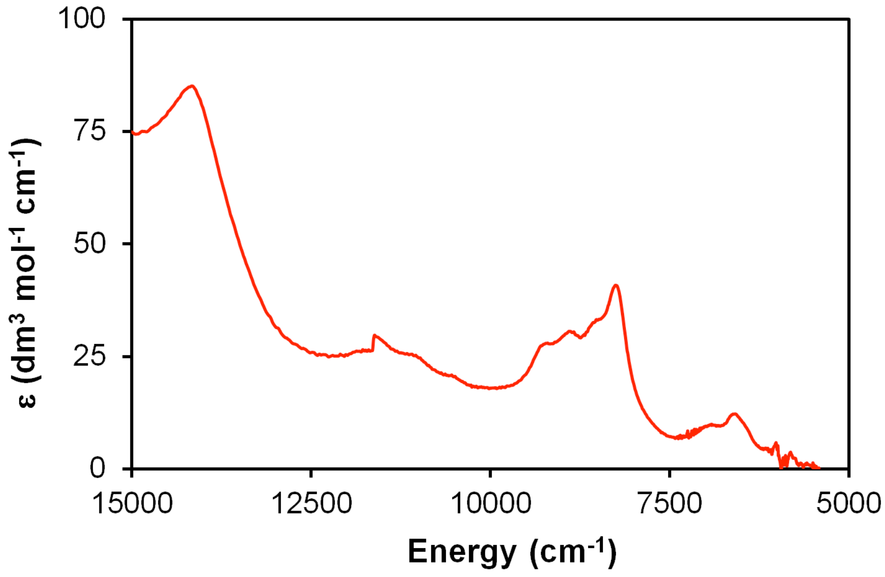

Figure S2. Zoom-in of the 5000-15000 cm^{-1} region of the UV/Vis/NIR spectrum of **2**.

Magnetism Data

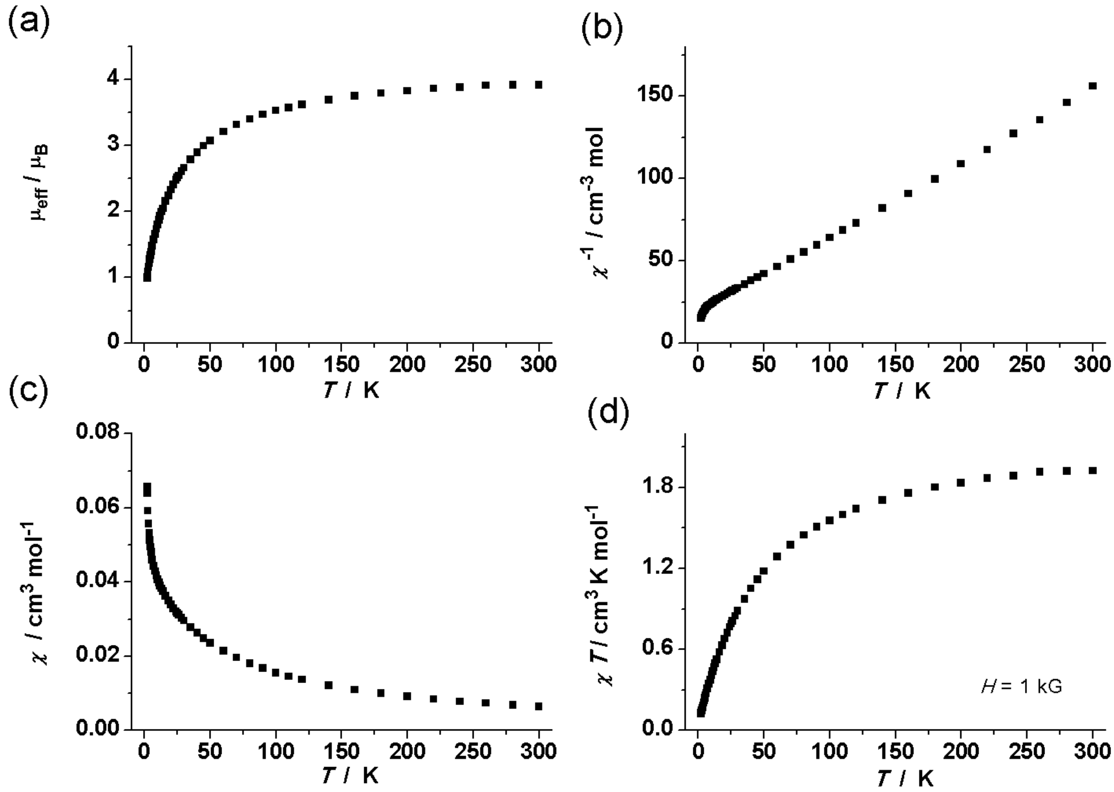

Figure S3. Variable-temperature SQUID data for: (a) μ_{eff} vs T, (b) $1/\chi$ vs T, (c) χ vs T, and (d) χT vs T for **2**.

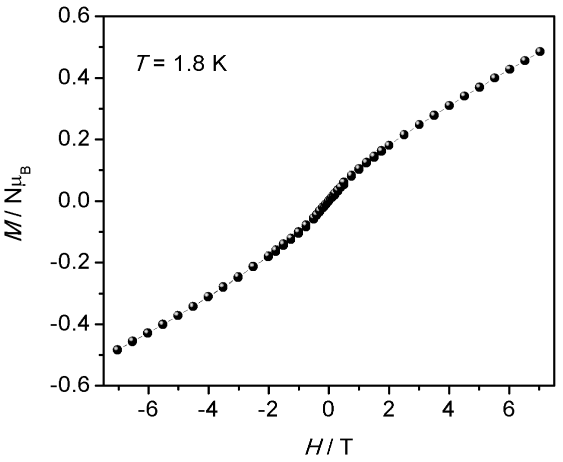

Figure S4. M vs H data for **2**.

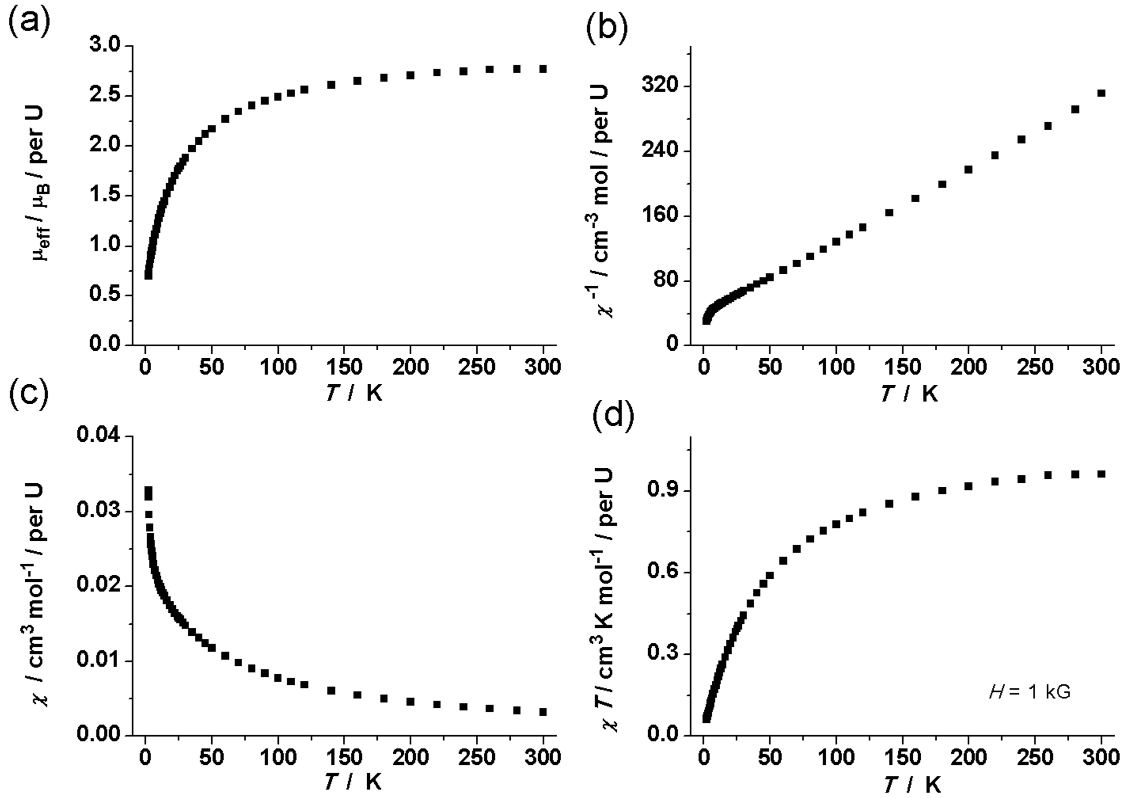

Figure S5. Variable-temperature SQUID data for: (a) μ_{eff} vs T, (b) $1/\chi$ vs T, (c) χ vs T, and (d) χT vs T peruranium ion in **2**.

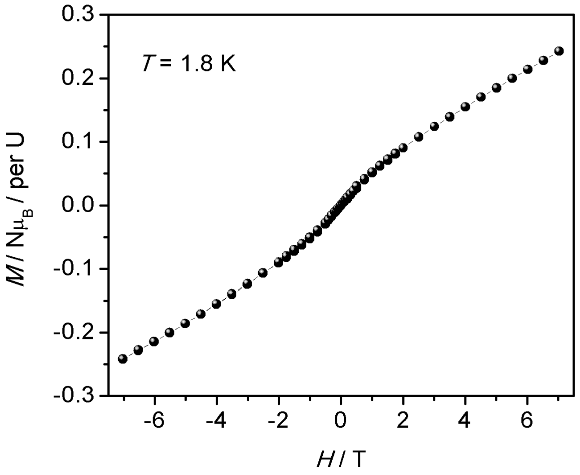

Figure S6. M vs H data per uranium ion in **2**.

Density Functional Theory Calculations

General

Unrestricted geometry optimisations were performed for the full model of **2** using coordinates derived from the X-ray crystal structure. No constraints were imposed on the structure during the geometry optimisation. The calculations were performed using the Amsterdam Density Functional (ADF) suite version 2012.01.[2,3] The DFT geometry optimisations employed Slater type orbital (STO) triple-ζ-plus polarisation all-electron basis sets (from the ZORA/TZP database of the ADF suite). Scalar relativistic approaches were used within the ZORA Hamiltonian for the inclusion of relativistic effects and the local density approximation (LDA) with the correlation potential due to Vosko et al[4] was used in all of the calculations. Gradient corrections were performed using the functionals of Becke[5] and Perdew.[6] MOLEKEL[7] was used to prepare the three-dimensional plot of the electron density.

Final Coordinates and Single Point Energy of 2 After Geometry Optimisation

1.C	-4.873949	-0.872082	-6.393979
2.C	-1.715692	0.929753	-6.319785

3.C	-1.731897	-0.384809	-5.512403
4.C	-4.785184	-0.252130	-4.980837
5.C	-0.306261	-0.720190	-5.042595
6.C	3.646240	0.566301	-4.902049
7.C	3.876468	-1.880316	-4.314423
8.C	-5.996242	-0.720791	-4.148071
9.C	-3.196339	-3.367817	-4.334187
10.C	3.571334	-0.470942	-3.764027
11.C	7.036930	-0.024608	-3.603203
12.C	-3.190274	2.127722	-3.444828
13.C	-2.084478	6.428338	-2.887117
14.C	-2.908923	-2.241538	-3.318615
15.C	0.138707	3.737366	-3.400909
16.C	-4.567543	2.530346	-2.934722
17.C	4.998898	2.776996	-2.587717
18.C	1.556956	5.563814	-2.458327
19.C	6.408040	-0.428272	-2.250361
20.C	1.375890	-4.179421	-2.315729
21.C	-0.317568	-5.986188	-1.944644
22.C	0.631403	4.380674	-2.090212
23.C	-3.704699	-2.472738	-2.022869
24.C	-1.679467	6.448255	-1.397096
25.C	-0.947573	7.775012	-1.092419
26.C	4.233046	1.809835	-1.655395
27.C	0.289090	-4.692239	-1.353682
28.C	-4.277507	4.051367	-1.000489
29.C	7.260897	0.158601	-1.104260
30.C	4.361792	-2.535284	-0.970501
31.C	2.974074	-6.702158	-0.338382
32.C	-5.580699	1.956447	-0.751143
33.C	-5.072685	0.573054	-0.364440
34.C	4.537300	2.211174	-0.199389
35.C	-3.190268	4.031797	0.063914
36.C	1.048172	-7.789485	0.862820
37.C	5.299518	-2.750329	0.208783
38.C	1.880970	-6.494415	0.732706
39.C	-1.977467	-5.731302	1.105564
40.C	1.418870	5.890713	0.998777
41.C	0.080724	5.127107	0.947763
42.C	-0.777129	-4.904185	1.608289
43.C	-5.433423	-2.328023	1.474211
44.C	4.011653	-3.950121	1.955826
45.C	-0.889790	5.692200	2.006422
46.C	2.538822	-3.791582	2.313557
47.C	-4.365001	-1.636775	2.348024
48.C	4.543569	-0.412720	2.406235
49.C	5.135257	-1.813460	2.497527
50.C	-6.203384	0.993134	2.601690
51.C	-6.263175	2.511539	2.877731
52.C	-0.503486	-5.219291	3.094410

53.C	-2.711247	2.463843	3.346057
54.C	-3.171882	1.012606	3.574202
55.C	1.967600	2.828467	3.764350
56.C	-4.324166	-2.288800	3.745039
57.C	-6.975892	0.246264	3.712265
58.C	4.419359	2.499646	4.235849
59.C	0.407891	-1.718992	4.561381
60.C	2.996130	1.987311	4.539318
61.C	0.914360	-0.296641	4.855418
62.C	-3.679698	0.883514	5.029313
63.C	3.994875	-0.754063	5.592144
64.C	3.678720	-2.224295	5.936212
65.C	0.615578	0.088199	6.319848
66.C	4.214421	0.035364	6.902648
67.H	-5.860294	-0.659044	-6.842017
68.H	-4.112243	-0.475296	-7.078166
69.H	-1.060258	0.832873	-7.202303
70.H	-2.710564	1.224803	-6.685231
71.H	-4.760876	-1.966503	-6.371177
72.H	-2.048539	-1.186138	-6.208857
73.H	-1.316740	1.761335	-5.719579
74.H	0.395107	-0.727898	-5.894629
75.H	3.040438	0.225210	-5.758852
76.H	-4.891004	0.839559	-5.123597
77.H	3.289371	-2.066066	-5.229444
78.H	4.671827	0.718379	-5.272151
79.H	-6.943231	-0.415198	-4.626058
80.H	-2.603826	-3.266473	-5.254795
81.H	4.936407	-2.006466	-4.584439
82.H	-3.172712	2.139826	-4.544291
83.H	-4.257816	-3.397730	-4.623215
84.H	6.546496	-0.511437	-4.457531
85.H	-0.243906	-1.702624	-4.554514
86.H	3.247348	1.543320	-4.595777
87.H	-6.019345	-1.817819	-4.064254
88.H	0.061310	0.028869	-4.324216
89.H	3.614209	-2.672197	-3.601797
90.H	-1.208646	6.554367	-3.540591
91.H	8.102738	-0.312077	-3.628739
92.H	6.992450	1.061962	-3.769752
93.H	-0.487457	4.426528	-3.984705
94.H	-2.954634	-4.350387	-3.895408
95.H	-2.776037	7.258351	-3.113768
96.H	0.998831	3.463095	-4.033777
97.H	4.836794	2.580248	-3.654143
98.H	-4.920443	3.468646	-3.402061
99.H	-5.999392	-0.319609	-3.124682
100.H	2.520108	-0.482421	-3.421001
101.H	-2.476030	2.913035	-3.141979
102.H	-2.579642	5.494295	-3.190688

103.H	-5.288505	1.743687	-3.185027
104.H	1.028368	6.306303	-3.074749
105.H	0.967234	-4.080551	-3.335279
106.H	-0.748170	-5.778205	-2.938458
107.H	2.407388	5.199465	-3.058022
108.H	-1.840422	-2.316904	-3.044570
109.H	-0.427106	2.813447	-3.232691
110.H	2.228648	-4.870655	-2.374699
111.H	6.083012	2.734700	-2.402653
112.H	4.682711	3.814646	-2.389988
113.H	6.503619	-1.527529	-2.189474
114.H	0.451605	-6.759863	-2.087573
115.H	-1.564271	8.632599	-1.414254
116.H	-4.789176	-2.378638	-2.174862
117.H	0.010587	7.848575	-1.627696
118.H	-3.936820	4.658146	-1.847604
119.H	4.904611	-2.705693	-1.911067
120.H	3.152861	1.979173	-1.825286
121.H	1.754145	-3.189336	-2.031289
122.H	2.535090	-6.974574	-1.309796
123.H	1.968143	6.088025	-1.587292
124.H	-1.111943	-6.420157	-1.325494
125.H	-6.494696	1.901762	-1.372153
126.H	-3.519951	-3.487074	-1.632067
127.H	-2.615862	6.478773	-0.811499
128.H	1.253482	3.629248	-1.567926
129.H	8.288386	-0.242697	-1.138338
130.H	7.341426	1.252411	-1.186780
131.H	-3.415166	-1.774267	-1.223656
132.H	-4.774365	0.049614	-1.287683
133.H	-0.514300	-3.930927	-1.341297
134.H	3.586013	-3.315963	-0.937055
135.H	-5.216799	4.498267	-0.622689
136.H	3.593652	-5.809401	-0.506304
137.H	3.650871	-7.524630	-0.049686
138.H	-0.740226	7.905147	-0.021255
139.H	0.479935	-8.006742	-0.053593
140.H	6.854656	-0.058732	-0.105415
141.H	-5.830008	2.511731	0.160714
142.H	4.296347	3.275949	-0.042845
143.H	-2.317120	-5.408540	0.112079
144.H	1.712082	-8.652914	1.043655
145.H	5.823611	-3.722604	0.146701
146.H	5.599705	2.078490	0.048195
147.H	-5.895494	-0.026384	0.051380
148.H	6.059871	-1.960684	0.209527
149.H	-3.079116	5.035124	0.498438
150.H	2.198758	5.395494	0.403965
151.H	1.329767	6.929629	0.645531
152.H	3.939198	1.650197	0.528499

153.H	-1.756897	-6.808655	1.053661
154.H	-5.347082	-2.051842	0.415966
155.H	-3.533758	3.392431	0.892301
156.H	0.332820	-7.747526	1.695278
157.H	4.105704	-4.699395	1.161279
158.H	2.400907	-6.372002	1.700429
159.H	0.304599	4.085266	1.243381
160.H	-2.831205	-5.610138	1.794488
161.H	-1.261764	6.694944	1.743895
162.H	-5.321113	-3.423875	1.528803
163.H	4.752216	-0.024212	1.394688
164.H	-1.086776	-3.842129	1.565554
165.H	1.785120	5.933220	2.038838
166.H	-6.769200	0.815232	1.669018
167.H	-6.458566	-2.095329	1.801731
168.H	-3.387630	-1.848440	1.873615
169.H	-1.758648	5.041909	2.165512
170.H	-5.710711	3.109525	2.138464
171.H	-2.214365	2.598876	2.376776
172.H	6.235564	-1.801868	2.388300
173.H	4.610875	-4.297424	2.818487
174.H	2.173484	-4.700550	2.812516
175.H	2.074400	2.683979	2.677844
176.H	-0.378099	5.786081	2.979163
177.H	-0.088727	-6.229156	3.239750
178.H	-7.307841	2.866969	2.876087
179.H	2.456791	-2.995003	3.071603
180.H	4.638229	2.453829	3.158082
181.H	5.073889	0.269586	3.085640
182.H	-3.549695	3.172452	3.405263
183.H	-2.273520	0.372451	3.483946
184.H	-1.441063	-5.172529	3.673818
185.H	0.188832	-4.501851	3.551048
186.H	4.903845	-2.238604	3.480830
187.H	0.471405	-1.973412	3.492719
188.H	-7.072563	-0.828463	3.507019
189.H	2.109383	3.903896	3.966603
190.H	-4.237321	-3.384149	3.645325
191.H	-5.843460	2.755211	3.865104
192.H	-1.981671	2.758669	4.118418
193.H	0.931694	2.576096	4.029701
194.H	-7.996536	0.655514	3.807974
195.H	0.321441	0.382371	4.215284
196.H	4.521849	3.556430	4.535466
197.H	-3.461984	-1.949834	4.335984
198.H	-5.234293	-2.090373	4.331645
199.H	-0.653750	-1.816405	4.842180
200.H	5.203122	1.935228	4.762986
201.H	-6.491754	0.355237	4.694132
202.H	0.961427	-2.485114	5.122569

203.H	-4.513329	1.574816	5.224844
204.H	3.507555	-2.849439	5.048095
205.H	-4.021301	-0.126811	5.285810
206.H	4.968232	-0.744454	5.067620
207.H	2.816725	2.157124	5.619246
208.H	-2.873692	1.149498	5.732658
209.H	-0.464701	0.005160	6.525169
210.H	0.914377	1.120198	6.553623
211.H	4.505547	-2.680455	6.507439
212.H	2.776649	-2.300128	6.562116
213.H	4.572788	1.058197	6.724200
214.H	1.129619	-0.576221	7.031355
215.H	4.968240	-0.470921	7.530807
216.H	3.293465	0.106850	7.500171
217.N	-2.795804	0.801918	-2.880011
218.N	-4.495161	2.663871	-1.463615
219.N	3.747013	-1.177047	-0.896560
220.N	-1.917228	3.508508	-0.514135
221.N	-3.917068	0.695500	0.568658
222.N	1.752881	-3.443459	1.095970
223.N	4.505940	-2.650581	1.453196
224.N	3.073305	-0.453533	2.669113
225.Si	-3.053965	-0.493346	-4.105460
226.Si	4.494397	-0.051824	-2.110014
227.Si	-0.726032	4.850050	-0.795103
228.Si	0.812822	-4.872496	0.496996
229.Si	-4.404784	0.301285	2.269034
230.Si	2.731848	0.080813	4.355360
231.P	0.552513	0.794034	-1.676350
232.P	-0.135084	-1.238995	-1.267988
233.P	1.218310	1.472055	0.298438
234.P	-0.950156	-1.206513	0.732318
235.P	-0.310609	0.650619	1.624789
236.U	-2.133132	1.256958	-0.745713
237.U	2.157999	-1.223911	0.727562

Energy: -1274.50108063 eV

References

1. D. M. King, F. Tuna, E. J. L. McInnes, J. McMaster, W. Lewis, A. J. Blake, S. T. Liddle, *Science* **2012**, *337*, 717.

2. C. Fonseca Guerra, J. G. Snijders, G. te Velde E. J. Baerends, *Theor. Chem. Acc.* **1998**, *99*, 391.

3. G. te Velde, F. M. Bickelhaupt, S. J. A. van Gisbergen, C. Fonseca Guerra, E. J. Baerends, J. G. Snijders T. Ziegler, *J. Comput. Chem.* **2001**, *22*, 931.

4. S. H. Vosko, L. Wilk, M. Nusair, *Can. J. Phys.* **1980**, *58*, 1200.

5. A. D. Becke, *Phys. Rev. A.* **1988**, *38*, 3098.

6. J. P. Perdew, *Phys. Rev. B.* **1986**, *33*, 8822.

7. S. Portmann, H. P. Luthi, *Chimia* **2000**, *54*, 766.
